# Eicosanoid Release Is Increased by Membrane Destabilization and CFTR Inhibition in Calu-3 Cells

**DOI:** 10.1371/journal.pone.0007116

**Published:** 2009-10-22

**Authors:** Florence Borot, Diane-Lore Vieu, Grazyna Faure, Janine Fritsch, Julien Colas, Sandra Moriceau, Maryvonne Baudouin-Legros, Franck Brouillard, Jesus Ayala-Sanmartin, Lhousseine Touqui, Marc Chanson, Aleksander Edelman, Mario Ollero

**Affiliations:** 1 INSERM, U845, Université Paris Descartes, Faculté de Médecine Paris Descartes, Paris, France; 2 Institut Pasteur, Unité d'Immunologie Structurale, CNRS, URA 2185, Paris, France; 3 CNRS, UMR7203, Groupe N. J. Conté, Laboratoire des BioMolécules, Paris, France; 4 Institut Pasteur, Unité de Défense Innée et Inflammation, INSERM, U874, Paris, France; 5 Laboratoire d'Investigation Clinique III, Hôpitaux Universitaires et Faculté de Médecine, Genève, Switzerland; LMU University of Munich, Germany

## Abstract

The antiinflammatory protein annexin-1 (ANXA1) and the adaptor S100A10 (p11), inhibit cytosolic phospholipase A2 (cPLA2α) by direct interaction. Since the latter is responsible for the cleavage of arachidonic acid at membrane phospholipids, all three proteins modulate eicosanoid production. We have previously shown the association of ANXA1 expression with that of CFTR, the multifactorial protein mutated in cystic fibrosis. This could in part account for the abnormal inflammatory status characteristic of this disease. We postulated that CFTR participates in the regulation of eicosanoid release by direct interaction with a complex containing ANXA1, p11 and cPLA2α. We first analyzed by plasmon surface resonance the in vitro binding of CFTR to the three proteins. A significant interaction between p11 and the NBD1 domain of CFTR was found. We observed in Calu-3 cells a rapid and partial redistribution of all four proteins in detergent resistant membranes (DRM) induced by TNF-α. This was concomitant with increased IL-8 synthesis and cPLA2α activation, ultimately resulting in eicosanoid (PGE2 and LTB4) overproduction. DRM destabilizing agent methyl-β-cyclodextrin induced further cPLA2α activation and eicosanoid release, but inhibited IL-8 synthesis. We tested in parallel the effect of short exposure of cells to CFTR inhibitors Inh172 and Gly-101. Both inhibitors induced a rapid increase in eicosanoid production. Longer exposure to Inh172 did not increase further eicosanoid release, but inhibited TNF-α-induced relocalization to DRM. These results show that (i) CFTR may form a complex with cPLA2α and ANXA1 *via* interaction with p11, (ii) CFTR inhibition and DRM disruption induce eicosanoid synthesis, and (iii) suggest that the putative cPLA2/ANXA1/p11/CFTR complex may participate in the modulation of the TNF-α-induced production of eicosanoids, pointing to the importance of membrane composition and CFTR function in the regulation of inflammation mediator synthesis.

## Introduction

Among the multisystemic clinical manifestations of cystic fibrosis (CF), an abnormal inflammatory condition at the airways represents one of the most prominent morbidity factors [1]–[3], resulting in bronchiectasis and respiratory insufficiency. The origin and nature of this response embodies a controversial subject. On the one side, many authors consider it secondary to recurrent infections and airway colonization by opportunistic pathogens [4]–[6]. On the other side, a growing body of evidence indicates that inflammation and infection in CF can be dissociated, and that a basal inflammatory status preexists to pathogen infections [7]–[9]. Reduced macrophage phagosome acidification related to defective Cl^−^ conduction has been reported as a potential cause of recurrent infections associated with chronic inflammation in CF [10], although this point has recently been contested [11]. It has also been suggested that increased inflammation and *Pseudomonas aeruginosa* colonization in CF could be secondary to intestinal malnutrition and decreased production of the anti-inflammatory cytokine IL-10 [12]. Conversely, there is evidence that proinflammatory cytokines can regulate CFTR expression [13], [14].

The expression of annexin-A1 (ANXA1) is strongly diminished in nasal cells from CF patients bearing codon stop and different non-sense mutations, including F508del –the most frequent [15]-, as well as in lung, intestine, and pancreas of CFTR knockout-mice ([15], [16] and unpublished observations). ANXA1 has been long known to be related to the regulation of inflammation, in particular by inhibition of the cytosolic phospholipase A2α (cPLA_2_), a member of the IV-A group of phospholipases A2 [17], [18]. Since cPLA2α releases the acyl chain at the sn-2 position of phospholipids leading to a subsequent synthesis of eicosanoids, the observed decrease in the expression of ANXA1 has been suggested to be at the start point of this cascade [16], [19]. The latter constitutes a key pathway in the onset and regulation of proinflammatory responses, including those altered in CF, as some studies have reported increased levels of eicosanoids in bronchoalveolar fluid from CF patients [20] and in the supernatant of epithelial cells expressing the F508del mutation of CFTR [21]. The molecular nature of cPLA2α inhibition is not precisely known. At least two proteins are candidates for regulation of this activity: the already mentioned ANXA1 and the adaptor protein S100A10 (p11), which forms a complex with another member of the annexin family, ANXA2, and coimmunoprecipitates with CFTR [22]. Moreover, annexins present a sequence homology with 30 aminoacids of the NBD1 domain of CFTR [23] including F508, suggesting that annexins and NBD1 may bind the same partners. p11 has been found to interact *in vitro* with cPLA2α and to inhibit its activity in both Beas 2B and MDCK cells [24], [25]. The inhibitory effect of ANXA1, initially suggested to respond to an enzyme inhibition-like process, was attributed to direct binding to phospholipids, which would prevent access of cPLA2α to its substrate [26]. Nevertheless, the high concentrations of Ca^2+^ required for inhibition suggested that the inhibitory mechanism of cPLA2α is other than a substrate-depletion phenomenon [27]. A recent work suggests a blockage by ANXA1 of cPLA2α translocation to the membrane as the putative mechanism of inhibition [28], most likely by direct specific binding to the cPLA2α-C2 domain [29].

cPLA2α activity can also be regulated by modulation of sphingolipids and cholesterol in CHO-2B cells [30]. This observation raises the potential role of detergent resistant membrane microdomains (DRM or lipid rafts) in the signaling of some innate immunity and inflammatory processes [31], [32]. The involvement of membrane microdomains in cPLA2α proinflammatory activity inflammation in the context of CF has never been questioned. It has recently been demonstrated a differential distribution of CFTR within membrane microdomains in CFTR-transfected MDCK cells [33], [34]. In basal conditions, mature CFTR is mostly present in detergent soluble fractions (non-DRM, non-rafts), with a minor proportion present in detergent-insoluble microdomains (DRM, rafts). Conversely, upon pathogen infection normal CFTR, unlike the mutated protein, redistributes into DRMs triggering defense reactions against the pathogen, including uptake and apoptosis of host cell [34]. This is consistent with earlier reports indicating that the response to pathogens would be associated with the presence of CFTR in a ceramide-rich environment [35]. Recently, Dudez et al, have demonstrated that wild-type CFTR, but not a truncated form lacking the C-terminal region, is recruited along with TNFR1 in DRM in response to TNF-α in MDCK transfected cells [36]. This would ultimately lead, *via* NF-κB activation, to the production of IL-8 [36]. In a recent report, it is claimed that CFTR localization in DRM of CFTR-transfected cells is key in limiting the activity of NF-κB and the synthesis of IL-8 [37]. However, the impact of the association of CFTR with DRM on the regulation of the eicosanoid signalling pathway has never been investigated. In this study we analyzed and confirmed the DRM association of CFTR, ANXA1, cPLA2α and p11 under proinflammatory conditions as well as the importance of membrane integrity in the synthesis and release of AA-derived eicosanoids, in an endogenous CFTR-expressing airway cell line.

## Methods

### Cell culture and treatments

Calu-3 cells (a human respiratory epithelial gland cell line from ATCC) were grown in Dulbecco's modified Eagle's medium (DMEM) supplemented with 10% foetal bovine serum at 37°C, and a 5% CO_2_ atmosphere as described previously [38]. In all experiments cells were grown to 80% confluence. For specific treatments, all chemicals were from Sigma except where indicated otherwise. To inhibit CFTR function, cells were maintained in serum-free medium for 24 hours and incubated with either 20 µM Inh172 or 20 µM Gly-101 (Calbiochem, La Jolla, CA). To stimulate the inflammatory response, cells were maintained in serum-free medium for 24 hours and incubated with TNF-α (100 U/mL, Alexis Biochemicals, Lausen, Switzerland) at different times. In order to disrupt DRM by depletion of membrane cholesterol, cells were preincubated for 24 h in serum-free DMEM followed by 1 h in the presence of 10 mM methyl-β-cyclodextrin (mβCD) at 37°C. The analogue αCD was used in the same conditions as mβCD. To inhibit cholesterol synthesis, cells were incubated for 48 h in the presence of 10 µM mevastatin. To inhibit sphingolipid synthesis, cells were incubated for 24 h with 20 µM fumonisin. Short incubations (10 min) with 10 or 50 µM exogenous arachidonic acid (AA) and 1-palmitoyl-sn-lysophosphatidylcholine (LPC) were performed in the presence of 0.2% (v/v) fatty acid-free bovine serum albumin. To inhibit cPLA2α activity cells were incubated with 15 µM N-{(2S, 4R)-4-(Biphenyl-2-ylmethyl-isobutyl-amino)-1-[2-(2, 4-difluorobenzoyl)-benzoyl]-pyrrolidin-2-ylmethyl}-3-[4-(2, 4-dioxothiazolidin-5-ylidenemethyl)-phenyl] acrylamide (pyrrolidine or PYR) (Calbiochem, San Diego, CA) for 45 min.

### Protein purification and preparation

Purified mouse NBD1 was generously provided by Dr. Philip Thomas (Southwestern University, Dallas, TX). Purification was performed as follows. A 70 mL inoculum of BL21-DE3 cells containing the SMT-3 fusion in the pET 28 expression system was grown overnight in LB medium at 37°C with kanamycin (50 ug/mL working concentration) present. Cells were induced with 0.179 g of IPTG, cooled to 15°C overnight, then harvested and pelleted at 4000 RPM at 4°C for 30 minutes. Each pellet was resuspended in 15 mL of lysis buffer (50 mM Tris, 100 mM L-Arginine, 50 mM NaCl, 5 mM MgCl_2_ hexahydrate, 12.5% Glycerol, 0.25 IGEPAL CA630, 2 mM 2-Mercaptoethanol, 2 mM ATP, pH 7.6). Suspensions were combined into 50 mL conical vials and lysed by sonication after adding lysozyme and incubating on ice for 30 minutes. The lysate was centrifuged at 40,000 g for 45 minutes to separate the soluble and insoluble fractions, and loaded into a pre-equilibrated 5 mL bed of Ni Sepharose 6 Fast Flow resin (GE Amersham). The column was equilibrated with 5 column volumes (CV) of Loading Buffer. During this step the elution tubes were prepared with 2 mM ATP and 2 mM 2-mercapto ethanol. The sample was loaded and bound to the column, and washed with 5 CV of washing buffer (20 mM Tris, 500 mM NaCl, 60 mM Imidazole, 12.5% Glycerol, pH 7.6). The sample was eluted in 5 CV of elution buffer (20 mM Tris, 250 mM NaCl, 400 mM Imidazole, 12.5% Glycerol, pH 7.6). Samples were taken for SDS PAGE analysis, and pooled together for concentration in a Beckman Coulter Allegra 6R centrifuge with a swinging bucket rotor, using the Amicon Ultra 15 30,000 MWCO centrifugal filters (Millipore). The protein was concentrated using 10-minute spins at 4000 rpm at 4°C. The SMT-3 fusion was cleaved off of NBD1 by using a 1∶1000 dilution of ubiquitin-like protease on ice for 1 hour. The protein was filtered using a Nalgene 0.22-micron syringe filter and injected onto a Hi Load 16/60 Superdex S200 prep grade gel filtration column (GE Amersham), and ran in S200 buffer (50 mM Tris, 150 mM NaCl, 5 mM MgCl_2_ hexahydrate, 2 mM ATP, 2 mM 2-mercapto ethanol, 12.5% glycerol, pH 7.6). The void volume fractions were rejected and the protein was loaded back onto the Ni Affinity column to remove the his-tagged SMT-3. The flow through was collected and concentrated in the same manner as before, except in a 10,000 MWCO Amicon Ultra 15 (Millipore). The protein was filtered again and injected onto the Superdex gel filtration column for buffer exchange. The flow through was collected and analyzed in a 10% SDS polyacrylamide gel to check for purity The sample was prepared by making a dilution of protein in S200 buffer into phosphate buffer (20 mM Na_2_PO_4_, 150 mM NaCl, 12.5%, Glycerol, 1 mM DTT pH 7.4) to give a 5 µM final concentration.

Purification of p11 protein was performed as previously published [39]. The p11 expression vector derived from pET-23a was kindly provided by Dr. Volker Gerke (Münster, Germany). Briefly, B834 (DE3) Escherichia coli cells were cultured in LB medium containing 100 µg/mL of ampicilin at an optical density of 0.6, measured at 600 nm, and then expression of p11 was induced by addition of 1 mM IPTG for 4 h at 37°C. Cells were pelleted and resuspended in lysis buffer (100 mM Tris pH 7.5, 200 mM NaCl, 10 mM MgCl_2_, 2 mM DTT) with protease inhibitors. Then they were lysed by sonication. After centrifugation for 15 min at 10 000 rpm in a SS34 Sorvall rotor, the supernatant was precipitated by (NH_4_)_2_SO_4_ at 50% saturation. The mixture was centrifuged at 15 000 rpm for 20 min, and the supernatant was applied onto a Butyl-Sepharose column equilibrated with the same buffer. p11 was eluted by a linear gradient of (NH_4_)_2_SO_4_ from 50% to 0% and recovered in the last fractions. After dialysis against 10 mM imidazole, pH 7.4, 1 mM EGTA, 0.1 mM EDTA, and 1 mM DTT buffer, the protein was applied onto a DEAE cellulose column equilibrated in the same buffer. The flow through containing p11 was dialyzed against PBS containing 1 mM DTT and concentrated with Centricon 3 (Amicon), and stored at −20°C in aliquots. At the end of purification, the p11 protein was more than 98% pure as judged by SDS−PAGE.

Purified human cPLA2α were generously provided by Dr. Michael H Gelb (University of Washington). Bovine ANXA1 was purchased from GenWay (San Diego, CA).

### Surface Plasmon Resonance (SPR)

Protein-protein interactions were studied in real time using a SPR Biacore 2000 system and CM5 sensor chips (Biacore AB, Uppsala, Sweden). NBD1 was covalently immobilized *via* primary amino groups onto the sensor chip surface as follows; the carboxymethylated dextran matrix was activated with 35 µl of EDC/NHS (1/1) mixture, 10 µl of NBD1 at a concentration of 50 µg/ml in 10 mM sodium acetate, pH 5.0, was injected and unreacted groups were blocked with 35 µl of ethanolamine (pH 8.5). A separate flow channel on the same sensor chip, reserved for control runs, was subjected to a blank immobilization by preparing it in the same way but without NBD1. The running and dilution buffer had the following composition: 50 mM Tris, 150 mM NaCl, 5 mM MgCl_2_ (pH 7.6), 0.005% P20, 1 mM DTT and 0.1 µM CaCl_2_. The interaction between either p11, ANXA1 or cPLA2/ANXA1 complex and the immobilized NBD1 was monitored by injecting different concentrations (30 µg/ml, 121 µg/ml and 243 µg/ml) of the mentioned purified proteins at 25°C with a flow rate of 30 µl/min, and following the refractive index changes at the sensor surface. The subsequent dissociation phase was followed after each association run by injecting the running buffer alone. In between injections, surfaces were regenerated by three washes with 20 µl of 5 mM NaOH followed by two washes with 20 µl of 1 M NaCl. For all SPR measurements, the recombinant domain was dialysed in the buffer with the desired NaCl concentration and pH, and centrifuged immediately before the runs to minimize possible effects from non-specific aggregation. All association and dissociation curves were corrected for non-specific binding by subtraction of control curves obtained from injection of the different analyte concentrations through the blank flow channel. The kinetic constants, k_on_ and k_off_, were calculated using the Biacore BIAEVALUATION 3.1 software (Biacore AB) assuming a simple two-component model of interaction. Experiments were performed 3 times.

### Preparation of membrane microdomains

The method was based on a previously described procedure [36]. Cells were cultured in 75 cm^2^ flasks, washed with ice cold PBS and scraped to obtain a cell pellet. Cell pellets were then lysed in 400 µL of ice-cold TEN buffer (25 mM Tris-HCl, 1 mM EDTA, 150 mM NaCl supplemented with 2 phosphatase inhibitors (1 mM NaVO_4_ and 1 mM NaF), and 1 tablet/10 mL of Complete-Mini protease inhibitor mixture (Roche Diagnostics), containing 1% Triton-X100, at 4°C for 20 min with agitation. Cell lysates were passed 10 times through a 21-gauge needle, and 400 µl of lysate were mixed with 800 µl of a 60% OptiPrep solution (Axis-Shield, Oslo, Norway) (40% final concentration). A three-step discontinuous OptiPrep gradient was prepared by layering 1.5 ml of 30% OptiPrep in detergent free lysis buffer and 500 µl of 5% OptiPrep on top. Gradients were centrifuged at 55,000 rpm for 2 h at 4°C in a Beckman XL-70 ultracentrifuge, using a swinging 55-Ti rotor. The top 1.2 ml were recovered and marked as DRM, as these reach their isopycnic point at the interphase 5/30% OptiPrep. The remaining 2 ml were collected and marked as non-DRM. Gradient fractions were subjected to protein concentration analysis by the DC method (Bio-Rad, Hercules, CA).

### Immunoblotting detection

An aliquot of each gradient fraction or pool was mixed with 1 volume of 5x Laemmli buffer. Samples were processed as previously described [40]. Briefly, they were heated at 37°C for 15 min, resolved by 8% SDS-PAGE, transferred onto PVDF membranes and blocked for 1 h with 5% non-fat milk diluted in TBS/Tween (0.1%). Blot membranes were hybridized using the following primary antibodies: monoclonal anti-CFTR 24.1 (1∶1000) (R&D Systems, Lille, France), monoclonal anti-CFTR MM13-4 (1∶1000) (Upstate Biotechnology, Guyancourt, France), monoclonal anti-CFTR M3A7 (1∶1000) (Abcam, Cambridge, UK), polyclonal anti-ANXA1 (1∶10000) (Zymed, San Francisco, USA), polyclonal anti-cPLA2α (1∶1000) (Santa-Cruz, Heidelberg, Germany), monoclonal anti-S100A10 (1∶5000) (BD Transduction, Erembodegem, Belgium), polyclonal anti-Caveolin-1 (1∶500) (Santa Cruz), monoclonal anti-Flotillin1 (1∶1000) (BD Transduction) and monoclonal anti-transferrin receptor (Zymed, 1∶1000). CFTR was detected by a horseradish peroxidase-coupled secondary antibody (AbCys, Paris, France) and incubation with the ECL-Plus solution (Amersham, Little Chalfont, UK). The rest of proteins were visualized by incubation with IRdye-coupled secondary antibodies and analysis by Odissey infrared imager (LI-COR Biosciences, Cambridge, UK). Densitometric quantification of bands corresponding was performed by the Quantity One software (Bio-Rad).

### cPLA2α activity

cPLA2α activity was estimated as the release of AA based on a method described elsewhere [41], cells were incubated for 18 h at 37°C with 0.5 µCi/mL [^3^H]-AA (Amersham) in DMEM containing foetal calf serum. Cells were then washed twice with PBS containing 0.2% fatty acid free BSA. Cells were subjected to the different treatments. At the end of incubation, cell supernatants were harvested and centrifuged at 10,000 g for 3 min at room temperature to eliminate cells and debris. The supernatants were counted for radioactivity by liquid scintillation.

### Eicosanoid production

For eicosanoid production assessment, cells were preincubated for 24 h in serum-free DMEM followed by specific treatments. At the end of incubation periods, supernatants were collected for analysis. The presence of PGE2 and LTB4 in supernatants was quantified using an enzymo-immunoassay analysis kit (Cayman Chemicals, Ann Harbor, MI). The assay is based on the competition between PGE2 (respectively LTB4) and PGE2 acetylcholinesterase conjugate (PGE2-tracer, respectively LTB4-tracer) for a limited amount of monoclonal PGE2 antibody (respectively LTB4 antibody). Because the concentration of the tracer is held constant while the concentration of eicosanoid varies, the amount of tracer that is able to bind to the monoclonal antibody is inversely proportional to the concentration of eicosanoid in the well. The eicosanoid-antibody complex binds to a goat polyclonal anti-mouse IgG that has been previously attached to the well. Then an acetylcholinesterase substrate (Ellman's Reagent) is added and the product of the reaction is read at 412 nm. The intensity is proportional to the amount of PGE2 (or LTB4). The assays were carried out according to the manufacturer's protocol, in triplicate. Eicosanoid quantification was performed using the software supplied by the kit manufacturer.

### IL-8 production

IL-8 was measured using an ELISA kit (CLB, Amsterdam, The Netherlands) in supernatants of Calu-3 cell cultures collected 3-hours following or not a 10 min pre-treatment with 100 U/ml TNF-α. Only assays having standard curves with a calculated regression line value >0.95 were accepted for analysis.

### Iodide efflux test

To assess CFTR Cl^−^ channel activity, iodide efflux was measured. from Calu-3 cells treated either with 10 µM forskolin plus 50 µM genistein or 20 µM CFTR Inh172, according to the protocol described by Hughes et al. [42]. Briefly, Calu-3 cells were cultured in 60 mm dishes until reaching 80–90% confluence. Cells were washed five times with 4 ml of loading buffer (136 mM NaI, 3 mM KNO_3_, 2 mM Ca(NO_3_)_2_, 20 mM Hepes, 11 mM glucose, pH 7,4) and incubated with this buffer for one hour at room temperature. Cells were gently washed fifteen times by adding 4 ml of efflux buffer (136 mM NaNO_3_, 3 mM KNO_3_, 2 mM Ca(NO_3_)_2_, 20 mM Hepes, 11 mM glucose, pH 7,4). Then, cells were incubated with 4 ml of fresh efflux buffer (drug-free or not) and at one-minute intervals it was collected and replaced by 4 ml of fresh efflux buffer, taking care not to expose cells to air. A iodide-selective electrode (ISE251, Radiometer Analytical SAS, France) connected to a pH meter (PHM250, Ion Analyzer, Radiometer Analytical SAS, France) were used to measure the amount of iodide released by cells at one minute intervals.

### Cholesterol and phospholipid analysis

Cholesterol and phospholipid content were monitored by thin-layer chromatography. The lipid-containing organic phase was obtained from gradient fractions by liquid-liquid extraction with six volumes of chloroform-methanol (2∶1, v/v), centrifuged at 800 g for 3 min, and the resulting lower phase aspirated. For cholesterol analysis, aliquots of 4 µl of the different samples and cholesterol standard were applied to HP-K plates (Whatman, Clifton, NJ), developed in chloroform-acetone (95∶5v/v), stained with the CuSO_4_ reagent, and developed by charring at 170°C. For phospholipid analysis, two sequential mobile phases were utilized: chloroform-triethylamine-ethanol-water (30∶30∶34∶8) and hexane-diethyl ether (100∶4.5). Images were captured by infrared detection in an Odissey apparatus (LI-COR Biosciences).

### Statistical analysis

Data are represented as percent of control values unless stated differently in the text. In all cases they are expressed as means ± SEM. Statistical significance was established by comparing original data by paired T-test when expressed as percent of control values, or by non-paired T-test in the other cases. p<0.05 was considered statistically significant. In all figures asterisks denote p<0.05 with respect to control, and unless indicated otherwise, n≥3.

## Results

### NBD1 fragment of CFTR interacts in vitro with S100A10 (p11)

**Figure 1:**
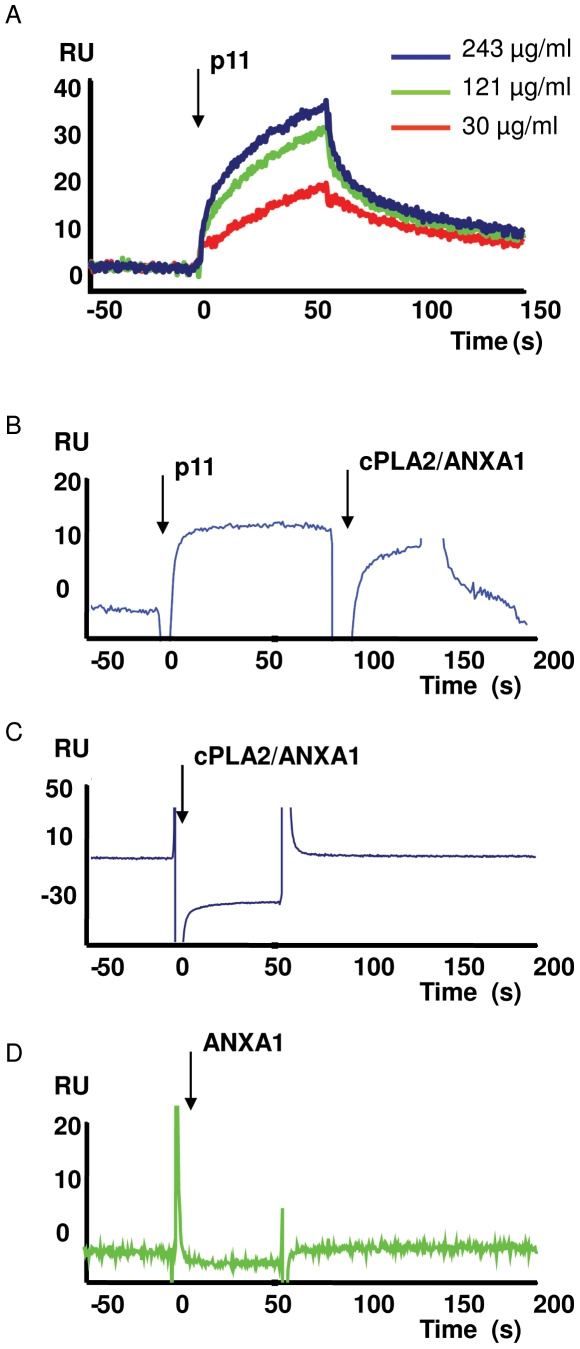
The direct interaction between NBD1 and p11 may connect CFTR to the cPLA2α/ANXA1 complex. A: SPR association curves of NBD1 and p11 (analyte) at serial concentrations of the latter. B: SPR association of NBD1 with p11 and cPLA2α/ANXA1 complex. p11 (400 µg/ml) and a preincubated cPLA2α/ANXA1 (100 µg/ml each) complex were sequentially co-injected as analytes (cPLA2α/ANXA1 was injected before dissociation of p11). C: Negative SPR association of NBD1 with a preincubated cPLA2α/ANXA1 complex (injected as analyte at 100 µg/ml each). D: Negative SPR association of NBD1 with ANXA1 (injected as analyte at 100 µg/ml).

Considering that cPLA2α interacts physically with ANXA1 and p11, we hypothesized that CFTR may be part of a putative complex including these proteins. Consequently, we aimed to establish a physical link between CFTR and any of the other components. In order to search for a direct interaction we performed SPR tests by immobilization of the NBD1 domain of CFTR and using the other three proteins as analytes. NBD1 domain was chosen as it possesses a structural homology with ANXA1 and may compete for the same protein partners, it contains the F508del mutation and it participates in channel opening by ATP binding. Our results indicate that only p11 shows a significant binding to NBD1 (Fig. 1A). Binding was dose-dependent and already significant at 30 µg/mL of analyte, which confirms a physical interaction *in vitro*. The association, dissociation, and apparent constants were respectively: k_on_(M^−1^s^−1^)  =  (1.4±0.2)×10^3^; k_off_(s^−1^)  =  (1.1±0.4)×10^−2^; K_d_^app^ = 7.8 µM. Injection of the cPLA2/ANXA1 complex before dissociation of p11 showed a significant binding (Fig. 1B), in contrast to the direct injection of cPLA2/ANXA1 on NBD1 in the absence of p11 (Fig. 1C). The drop in the baseline right after injection of cPLA2/ANXA1 reflects an artefactual effect of the buffer containing cPLA2 due to a pH change. Finally, as a negative control, ANXA1 fails to bind to NBD1 (Fig. 1D). These results suggest p11 as the link between CFTR and the cPLA2/ANXA1 complex.

### TNF-α stimulation of Calu-3 cells recruits CFTR complex in DRM

**Figure 2:**
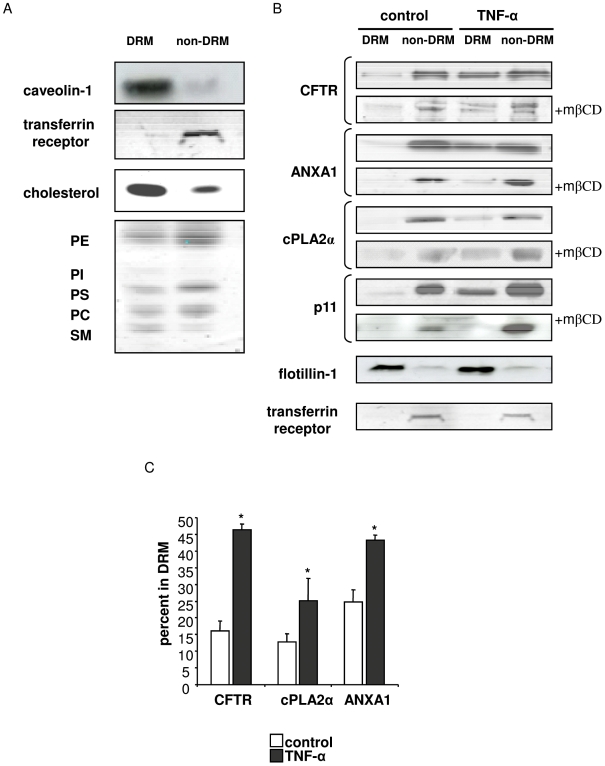
CFTR, ANXA1, cPLA2α and p11 are partially recruited in DRM by TNF-α. A: Characterization of detergent resistant and non-resistant fractions from Calu-3 cells. Cells were incubated in 1% Triton X-100 and subjected to OptiPrep gradient separation. DRM and non-DRM fractions were collected separately and subjected to western blotting analysis for Caveolin-1, transferrin receptor, and to cholesterol and phospholipid analysis (PE: phosphatidylethanolamine, PI: posphatidylinositol, PS: phosphatidylserine, PC: phosphatidylcholine, SM: sphingomyelin). Results are representative of at least 3 experiments. B: TNF-α recruits CFTR, ANXA1, cPLA2α and p11 in DRM. Calu-3 cells were treated with or without 100 U/mL TNF-α for 10 min, in the presence and absence of 10 mM mβCD, processed as in A and subjected to western blotting analysis of CFTR, ANXA1, cPLA2α and p11. Results are representative of at least 3 experiments. C: Densitometric quantification of DRM localization of CFTR, ANXA1 and cPLA2α. Western blot bands corresponding to DRM and non-DRM were quantified. Data are expressed as the percent of each protein present in DRM (means ±SEM, n≥3).

As we have previously reported on Calu-3 cells, CFTR is mostly present in detergent soluble cell membrane fractions, but it is significantly recruited in DRM upon TNF-α stimulation [36]. We wanted to establish the DRM distribution of ANXA1, cPLA2α and p11 in basal conditions in this cell line, which expresses endogenously CFTR. DRM and non-DRM fractions were characterized by the localization of positive and negative markers (Fig. 2A). Caveolin-1 was mainly present in DRM, like cholesterol, sphingomyelin and phosphatidylinositol. Transferrin receptor, a known negative marker of DRM, was mostly present in non-DRM, like phosphatidylcholine, phosphatidylethanolamine and phosphatidylserine (Fig. 2A).

By immunoblotting detection (see MethodSection, *Immunoblotting detection for details*), all four proteins (CFTR, ANXA1, cPLA2α, and p11) showed an essential distribution in non-DRM in basal conditions, with a minor but significant fraction localized to DRM (Fig. 2B and 2C). Following a short TNF-α stimulation (10 min), a significant recruitment of all four proteins in DRM was observed. This was partially prevented by the DRM-destabilizing agent mβCD (Fig. 2B). As positive and negative controls respectively, flotillin-1 and the transferrin receptor were not relocalized after treatment (Fig. 2B). Longer exposure to the stimulus (3 h) did not change substantially DRM localization (not shown), suggesting that the observed changes are rapid and independent from putative transcriptional effects of TNF-α, which are known to occur after longer stimulation periods [43]. These results indicate that the four proteins present a similar distribution pattern and response to TNF-α, and suggest that they may form a functional complex.

### TNF-α induces eicosanoid release

**Figure 3:**
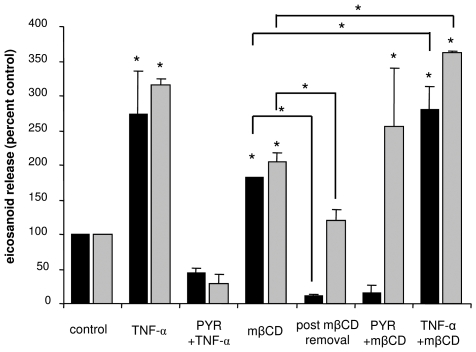
Effect of TNF-α and DRM destabilization on eicosanoid production. Calu-3 cells were treated with either 100 U/mL TNF-α for 10 min, 10 mM mβCD for 1 h, with or without preincubation with 15 µM pyrrolidine for 45 min, or with a combination of the different treatments. For combined treatments, TNF-α was added for the last 10 min of incubation. After incubation the supernatant was collected, either immediately or after 3 h of incubation in fresh medium, and subjected to ELISA for LTB4 and PGE2 determination. Results are expressed as percent of control values. Asterisks denote p<0.05 with respect to control, n≥3.

**Figure 4:**
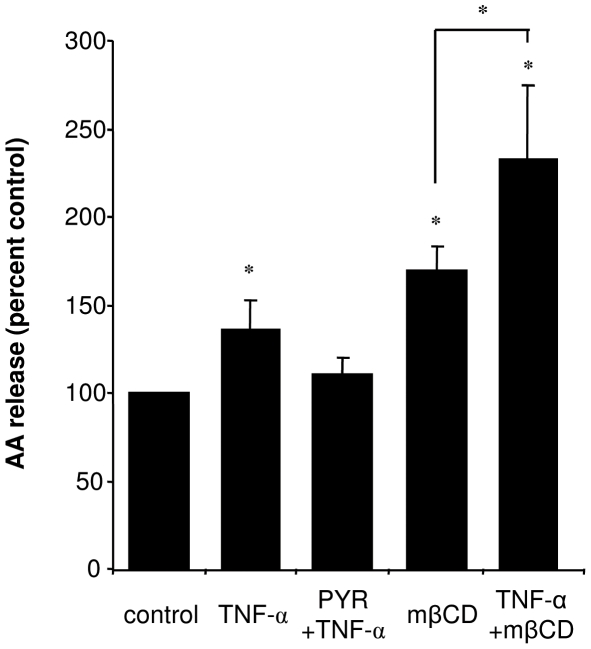
Effect of TNF-α and DRM destabilization on AA release. Calu-3 cells were incubated overnight with either ^3^H-labelled AA, treated with 100 U/mL TNF-α for 10 min, 10 mM mβCD for 1 h, with or without preincubation with 15 µM pyrrolidine for 45 min, or with a combination of the different treatments. For combined treatments, TNF-α was added for the last 10 min of incubation. After incubation, supernatants were collected and radioactivity measured by a scintillation counter. Results are expressed as percent increment with respect to control. Asterisks denote p<0.05 with respect to control unless indicated otherwise, n = 3.

Given the partial recruitment of the protein complex in DRM under the effect of TNF-α stimulation, we assessed eicosanoid and cytokine synthesis in the same conditions. Cells were treated with 100 U/mL of TNF-α for 10 min, and the release of PGE2 and LTB4 to incubation medium for 3 h was estimated. As shown in Figure 3, both lipid mediators were increased about 3-fold by TNF-α treatment (Fig. 3A). This effect was prevented by preincubation of cells with the cPLA2α inhibitor pyrrolidine, strongly suggesting that TNF-α induces eicosanoid release *via* cPLA2α activation. This was confirmed as Calu-3 cells released a significantly greater amount of radiolabelled arachidonic acid (AA) after TNF-α treatment than in control conditions, which was equally prevented by pyrrolidine (Fig. 4). AA release was an early event, as it was detected 10 min after TNF-α addition to the medium.

**Figure 5:**
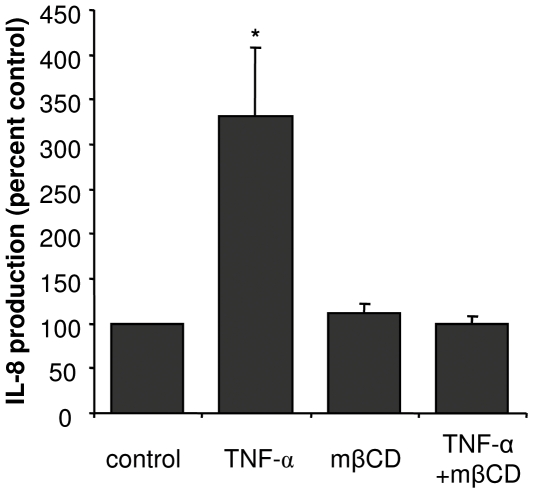
Impact of TNF-α and DRM destabilization on IL-8 release. IL-8 production by Calu-3 cells after proinflammatory stimulation, functional inhibition of CFTR and DRM disruption. Calu-3 cells were incubated with either 100 U/mL TNF-α for 10 min, 10 mM mβCD for 1 h, or with a combination of both treatments. After incubation, supernatants were removed and fresh DMEM medium containing fetal calf serum was added. After 3 h of incubation, supernatants were harvested for IL-8 determination. Results are expressed as percent of control values. Asterisks denote p<0.05 with respect to control, n≥3.

TNF-α has previously been shown to recruit CFTR along with TNFR1 to DRMs, which may be critical to regulate IL-8 release [36]. In a separate series of experiments we tested the effect of TNF-α treatment on IL-8 release in the same conditions as eicosanoid analysis. IL-8 was increased by more than 3-fold and prevented by incubation of cells with the cholesterol depleting agent mβCD (Fig. 5). These results suggest that DRM integrity is necessary to initiate the signaling events leading to NF-κB activation and IL-8 synthesis in Calu-3 cells.

### DRM destabilization inhibits TNF-α-evoked IL-8 production and induces eicosanoid release

Like TNFR1, cPLA2α activation by TNF-α could be a consequence of its relocalization to DRM. To address this point, we examined whether inhibition of cPLA2α recruitment by DRM destabilization has an impact on cPLA2α activity. For this purpose, we measured the release of eicosanoids in the medium after mβCD treatment. As shown in Figure 3, secreted LTB4 and PGE2 levels were increased by 2-fold after 1 h of mβCD treatment. This effect was not sustained in time, as the concentration of both eicosanoids in the culture medium 3 h after mβCD removal was significantly decreased (“post mβCD removal” in Fig. 3). Interestingly, pyrrolidine inhibited the release of LTB4 under these conditions, but not that of PGE2 (Fig. 3) suggesting that two different mechanisms are involved. When cells were treated by both mβCD and TNF-α, the increase in both eicosanoids was greater than in the presence of mβCD alone, but not greater than TNF-α alone (Fig. 3). We evaluated the release of radiolabelled AA in the same conditions, and observed a significant increase by mβCD and again a greater release when mβCD and TNF-α were combined (Fig. 4). This confirms the involvement of cPLA2α activity in mβCD effect, and suggests different mechanisms of activation by mβCD and TNF-α. This also indicates that DRM integrity is not necessary for cPLA2α activation.

In order to test whether the stimulatory effect of mβCD is exclusive of eicosanoid synthesis, we verified whether this drug alone has an impact on a different inflammatory mediator pathway. We chose to assess IL-8 synthesis, as DRM play a role in its regulation [36]. As shown in Figure 5, mβCD treatment did not change IL-8 release, which is consistent with its inhibiting effect on TNF-α-induced stimulation (Fig. 5), supporting the view that DRM integrity may be a rate limiting factor for IL-8 production (Fig. 5).

**Figure 6:**
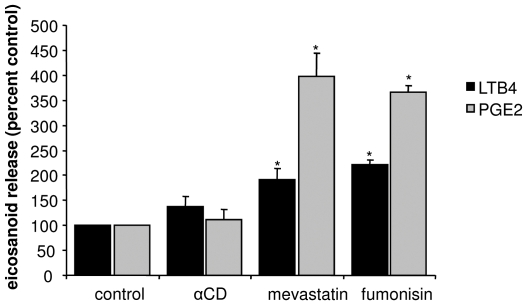
Effect of DRM disruption agents on eicosanoid production. Calu-3 cells were incubated with either, 10 mM αCD for 60 min, 10 µM mevastatin for 48 h, or 20 µM fumonisin for 24 h. After incubation the supernatant was collected and subjected to ELISA for LTB4 and PGE2 determination. Data are expressed as percent of control values.

To discard a potential non-specific effect of mβCD, we treated cells with its non sterol-extracting analog αCD. In addition, we induced DRM destabilization by several alternative mechanisms. These included both the decrease in membrane cholesterol content by the sterol synthesis inhibitor mevastatin, and the decrease in membrane sphingolipid content by the sphingolipid synthesis inhibitor fumonisin. Eicosanoid release levels were measured. As shown in Figure 6, eicosanoid production of cells treated with αCD was not significantly different from that of control cells. Conversely, the two alternative DRM-disrupting treatments led to a significant increase in LTB4 and PGE2 secretion.

These results confirm that DRM recruitment of CFTR, ANXA1, cPLA2α and p11 is not a necessary event in PGE2 and LTB4 release. Conversely, they suggest that membrane composition may play a role in the regulation of eicosanoid production.

### Short term Inh172 treatment of Calu-3 cells increases PGE2 and LTB4 release

**Figure 7:**
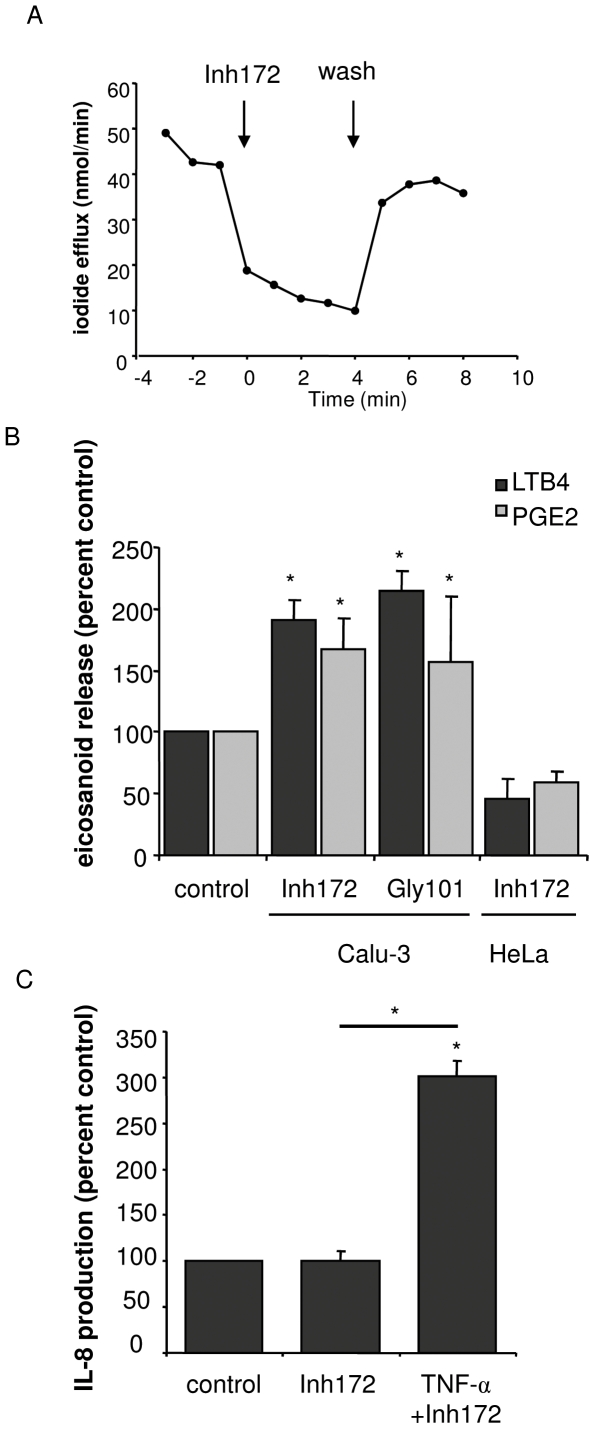
Effect of CFTR inhibition on eicosanoid, AA and IL-8 release. A: Iodide efflux (CFTR activity) measurements on Calu-3 cells. Effect of 10 min incubation with 10 µM Inh172 in Calu-3 cells (left). Effect of forskolin activation (right). Results are representative of three experiments. B: Calu-3 cells were treated with either 20 µM Inh172 or Gly-101 for 20 min. In a separate experiment, HeLa cells were treated with 20 µM Inh172 for 20 min. The supernatant was collected right after treatment and subjected to ELISA for LTB4 and PGE2 determination. C: Calu-3 cells were incubated with or without 20 µM Inh172 for 20 min. After incubation, supernatants were removed and fresh DMEM medium containing fetal calf serum was added. After 3 h supernatants were harvested for IL-8 determination. All results are expressed as percent of control values.

To examine the question of whether TNF-α-stimulated recruitment of CFTR, ANXA1 and cPLA2α in DRM, and eicosanoid production are associated with CFTR activity, Calu-3 cells were incubated for 20 min with two inhibitors of the chloride channel function of CFTR: Inh172 and Gly-101. Firstly, we verified whether CFTR is active in Calu-3 in our experimental conditions. Inh172 inhibited iodide efflux by 38% on average (Fig. 7A), indicating a significant basal activity of the channel. This activity was stimulated by forskolin (not shown). These results are in agreement to previous reports of CFTR activity in Calu-3 cells in non-stimulating conditions [44], [45].

**Figure 8:**
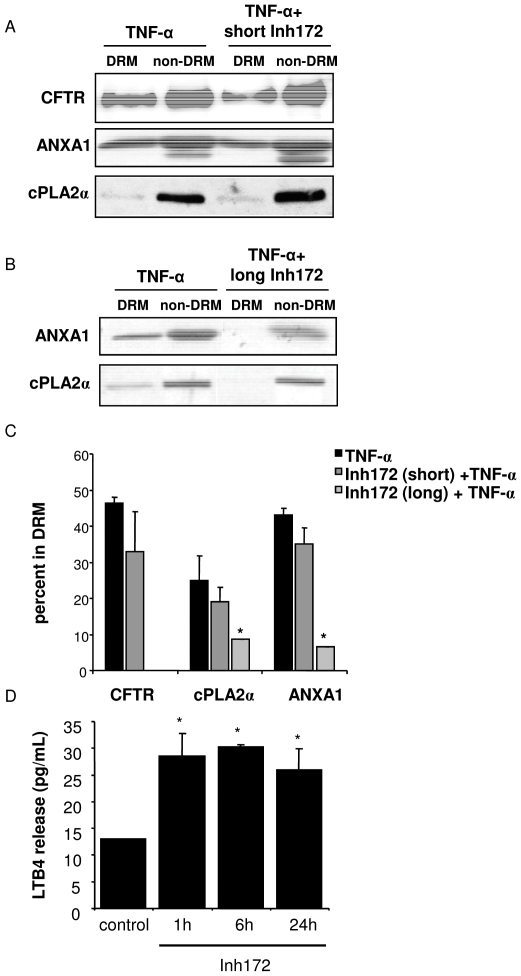
Effect of CFTR inhibition on DRM localization of ANXA1, cPLA2α and CFTR. A: Calu-3 cells were treated with 100 U/mL TNF-α for 10 min alone or after preincubation with 20 µM Inh172 for 20 min. After treatment, cells were incubated in 1% Triton X-100 and subjected to OptiPrep gradient separation. DRM and non-DRM fractions were pooled separately and subjected to western blotting analysis of CFTR, ANXA1, and cPLA2α. Results are representative of at least 3 experiments. B: Same as in A, except preincubation time with 20 µM Inh172 for 12 h. Western blot for CFTR is not shown. C: Densitometric quantification of DRM localization of CFTR, ANXA1 and cPLA2α. Western blot bands corresponding to DRM and non-DRM were quantified. Data are expressed as the percent of each protein present in DRM (means±SEM, n≥3). D: Calu-3 cells were incubated with 20 µM Inh172 for either 1 h, 6 h or 24 h. After incubation the supernatant was collected and subjected to ELISA for LTB4 determination.

Both inhibitors increased about 2-fold the secretion of LTB4 and PGE2 with respect to control (Fig. 7B) suggesting the involvement of CFTR function in eicosanoid synthesis and a relatively rapid effect of its inhibition. In a single control experiment, the non CFTR-expressing HeLa cells were treated with Inh172 in the same conditions as Calu-3, and no stimulation of eicosanoid release was obtained (Fig. 7B). Inh172 had no effect on basal and TNF-α-induced IL-8 secretion (Fig. 7C), which is consistent with the non-interference of CFTR inhibition with TNF-α-induced relocalization of ANXA1 and cPLA2α in DRM, as it is shown by western blot in Figure 8A and by densitometry in Fig. 8C, while Inh172 alone had no effect on DRM localization (not shown).

### TNF-α-induced DRM recruitment of ANXA1 and cPLA2α is inhibited by long exposure to Inh172

Longer exposure of Calu-3 cells to Inh172 has been associated with increased levels of IL-8 [46]. We tested whether a long inhibition of CFTR (12 h) had an effect on DRM recruitment. As shown in Figures 8B and 8C, significantly less ANXA1 and cPLA2α were found in DRM in these conditions, after TNF-α treatment, as compared to TNF-α treatment alone. It must be noted that CFTR levels were too weak to be detected, even in non-DRM, after long term incubation with Inh172 (not shown), suggesting that CFTR expression was diminished. This raised the question of whether long-term Inh172 was able to modify eicosanoid production. We analyzed the time-dependent release of LTB4 in inhibiting conditions (Fig. 8D). The maximum release was reached at 1 h, similar to that at 20 min, indicating that no further eicosanoid synthesis occurs beyond this point. These results suggest that the two reported effects of Inh172 -stimulation of eicosanoid release and inhibition of DRM recruitment- respond to dissociated mechanisms.

## Discussion

The aim of this work was to examine the hypothesis that CFTR interacts with major protein(s) of the cPLA2α /eicosanoid pathway and that it may function as a modulator of eicosanoid production. For this purpose, the potential role of a putative CFTR/cPLA2α /ANXA1/p11 complex in the context of DRM and eicosanoid and cytokine synthesis was investigated. The results show that the connection between membrane microdomains and CFTR/cPLA2α/ANXA1/p11 is complicated but fundamental for eicosanoid and IL-8 production during the acute phase of TNFα-induced inflammation (10–20 min). Interestingly, the chloride channel function of CFTR seems to regulate eicosanoid synthesis in basal conditions.

We confirm in Calu-3 cells the previous observations obtained in other models that short TNF-α stimulation induces CFTR recruitment at DRM [34], [36]. In addition, we show for the first time a synchronous dynamics of the AA-releasing enzyme cPLA2α, and the cPLA2α-regulatory proteins ANXA1 and p11 (S100A10), and prove that membrane integrity participates in the regulation of eicosanoid synthesis and release. We demonstrate that CFTR interacts *in vitro* with the cPLA2α/ANXA1 complex *via* NBD1/p11 binding. The pathway examined in our study, involving AA release and subsequent eicosanoid production, could play a fundamental role in the homeostasis of inflammation in epithelial cells, both by the direct bioactive properties of LTB4 and PGE2 on the surrounding cells and tissues, and by representing a regulatory circuit of the NF-κB and cytokine expression path, as in two different models ANXA1 has been shown to regulate the synthesis of proinflammatory cytokines TNF-α and IL-1β [47], [48].

Our central premise was the fact that TNF-α induces, at a short incubation time, both recruitment of certain proteins in DRM [36] and activation of cPLA2α [49]. It is tempting to hypothesize that one of the phospholipid cleavage products, namely AA, LPC or their downstream derivatives, might be responsible for CFTR, ANXA1, cPLA2α and p11 recruitment into DRM. We tested the effect of exogenous AA and LPC, and in both cases the result was negative; conversely, preliminary tests with the cPLA2 inhibitor pyrrolidine gave similar results (not shown). It must be considered that TNF-α-induced DRM recruitment is a fast event (10 min) and it is unlikely that downstream products, such as eicosanoids, participate in the mechanism. DRM recruitment could also be explained from an alternative hypothesis. As cPLA2α activity participates in the turnover of membrane phospholipids, a sudden activation would result in a rapid lipid remodeling, leading to a change in the affinity of certain proteins for the surrounding environment and subsequent relocalization to cholesterol-containing DRM domains. Whether this phenomenon can happen in cell membranes and within 10 min remains to be elucidated.

Concerning TNF-α stimulation of cPLA2α activity, several studies show that two different pathways could be involved [49]. The first entails the activation of MAPK and subsequent phosphorylation of cPLA2α, and occurs *via* activation of TNFR1. The second pathway would be independent from phosphorylation and would involve a raise in intracellular Ca^2+^, leading to translocation of cPLA2α to the activation sites, mainly the endoplasmic reticulum, the Golgi apparatus and the nuclear envelope. An early work [50] indicates that the first pathway would be activated by default. Our results suggest that both events triggered by short exposure to TNF-α, i.e. cPLA2α activation and DRM recruitment of CFTR, ANXA1, cPLA2α and p11, may occur independently of one another, as DRM destabilization does not inhibit AA release induced by the cytokine (Fig. 5).

A logical question prompted by our observations on DRM relocalization of proteins was that of the impact of DRM disruption on cPLA2α activity and eicosanoid synthesis. This led to the remarkable and surprising finding that all the treatments tested involving depletion of either cholesterol or sphingolipids –two lipid classes enriched in DRM- result in increased LTB4 and PGE2 synthesis. Interestingly, long term treatments (mevastatin and fumonisin) have a greater effect on PGE2 than on LTB4, as compared to mβCD. The former may have an impact on COX-2 expression, leading to increased PGE2 production. This has already been evaluated in macrophages, but while fumonisin enhances COX-2 expression in the presence of LPS [51], statins are mainly inhibitory [52]. Our results support the hypothesis that the presence of CFTR, ANXA1, cPLA2α and p11 –all four altered in terms of function or expression in CF [16]- in DRM would limit eicosanoid synthesis. Nevertheless, even though the increased AA release after mβCD treatment would suggest the regulation of this synthetic mechanism to take place at the cPLA2α checkpoint, our findings do not discard the possibility that mβCD has a direct impact on cyclooxygenase and lipooxygenase activities. In particular, the non-inhibition by pyrrolidine of mβCD-induced PGE2 release, strongly suggests a direct activation of cyclooxygenase function. In fact, it has been reported that mβCD induces COX-2 epression and activation in a macrophage cell line, but at a much longer incubation time [53]. An alternative hypothesis for the shorter term effect of cholesterol depletion could involve PKC and Src triggering and subsequent activation of COX-2, as both events have been shown independently in other cell types [54], [55]. Exploration of these or other hypotheses is warranted in order to understand the effects of DRM disruption. The fact that TNF-α and mβCD together seem to exert a greater effect than each stimulus alone, which is especially clear in terms of AA release, suggests a deregulation of the system, probably due to changes in membrane composition that could alter protein-protein interactions. Nonetheless, it is noteworthy to remark that DRM destabilization by mβCD, mevastatin and fumonisin, and the parallel increase in eicosanoid production points to the importance of membrane composition in the inflammatory response.

It could also be hypothesized that DRM localization would reduce the availability of cPLA2α substrate, since polysunsaturated fatty acid-containing phosphatidylcholine is mostly present in non-DRM (Fig. 2A and [56]). Another possibility is that DRM recruitment leads to the formation of a functional complex in response to the inflammatory stimulus that would limit the catalytic activity of cPLA2α by direct binding of ANXA1 and/or p11. The synchronous recruitment in DRM of the four proteins seems to be in agreement with this hypothesis. However, the small proportion of cPLA2α that is relocalized to DRM does not seem to account for a major reduction in its catalytic activity in any of the two cases.

An alternative possibility could be that the putative complex triggers an unknown regulatory mechanism. The answer may reside in the identification of the other partners that compose the complex. An obvious question that could be raised is if the integrity of the putative complex would depend on CFTR function and/or on TNF-α stimulation. In this study we demonstrate for the first time the direct interaction *in vitro* of NBD1 and p11, which could constitute the link between CFTR and ANXA1/cPLA2α. Ongoing studies in our laboratory pursue the identification of additional partners of CFTR. To this respect it cannot be excluded the existence of a functional short-lived dynamic complex which we have already hypothesized [15], [57].

An unexpected finding was the stimulatory effect of CFTR inhibition on eicosanoid production in basal conditions, especially considering that LTB4 and PGE2 were found already increased after 20 min of treatment, as compared to the longer period of time (3 h) necessary for their accumulation in the medium after TNF-α stimulation. The fact that a similar effect was obtained with two independent inhibitors (Inh172 and Gly-101), and that no stimulation was found in the non-CFTR expressing cells HeLa (Fig. 7B), give credence to the results and strongly suggests a mechanism involving the chloride channel function of CFTR. Nevertheless, these results must be taken cautiously, as in our experiments cells were not treated with the CFTR activation cocktail. Although Calu-3 cells show a modest but significant CFTR activity in basal conditions (Fig. 7A), in agreement with previous reports[44], [45], we cannot exclude an effect of Inh172 and Gly-101 independent of CFTR inhibition. Longer exposure (up to 24 h) of cells to Inh172 did not change the extent of LTB4 release (see Fig. 8D). This suggests that no further synthesis of LTB4 occurs beyond 20 min of incubation. However, one would expect that LTB4 would be taken up and metabolized by cells within 24 h. The fact that LTB4 levels do not return to control values could be due to a raise in the background production as a consequence of CFTR inhibition. Hence, the link between CFTR function, cytokine and lipid mediator production opens an exciting field for future research.

We also tested whether CFTR function would be necessary for recruitment. This hypothesis was based on our recent results reporting an absence of TNF-α-triggered recruitment of a TRLdel non-functional mutant of CFTR in MDCK cells [36]. Short incubation (20 min) of cells with Inh172 did not show any effect on relocalization (see Fig. 8A and 8C). The experimental conditions used –incubation time and inhibitor concentration-, should be sufficient to assure total inhibition of CFTR chloride channel activity [58]. As we show the presence of a significant basal activity of CFTR in Calu-3 cells, it can be concluded that CFTR function as a chloride channel is not linked to DRM relocalization of cPLA2α and ANXA1. In fact, we have addressed this point by testing protein recruitment in DRM in conditions of CFTR activation with forskolin, IBMX and cAMP, and no differences were observed as compared to basal settings (data not shown). These results are in agreement with a recent report suggesting no relationship between CFTR chloride channel activity and DRM localization [36], [59], and suggest that an alternative function of CFTR may be involved. However, long term (12 hours) inhibition of CFTR significantly decreased the recruitment in DRM of both cPLA2α and ANXA1 (Fig. 8B and 8C). This may be due to an insufficient amount of CFTR at the plasma membrane, since we found that long exposure of Calu-3 cells to Inh172 leads to decreased detection of CFTR. This might obey to decreased expression of CFTR, as our preliminary results show that the amount of CFTR transcripts is diminished after 18 h of treatment of Calu-3 cells by Inh172. Alternatively, the long term effect of Inh172 treatment points towards a scenario in which other functions of CFTR, distinct from the chloride channel activity [15], [36], may play a role in TNF-α-induced DRM relocalization.

In both MDCK and CFBE41o cells, it has been demonstrated a link between this relocalization in DRM and the regulation of the TNF-α-induced signaling pathway leading to NF-κB activation and cytokine synthesis [36], [37]. In Calu-3 cells, we have found an equivalent function, as TNF-α stimulates IL-8 secretion, which is inhibited by mβCD. In epithelial cell lines and primary cultures, longer inhibition of CFTR by Inh172 (3 to 5 days) results in increased production of IL-8 [46]. This appears to be in disagreement with our observation that long term Inh172 prevented DRM recruitment, though the conditions and models were different.

**Figure 9:**
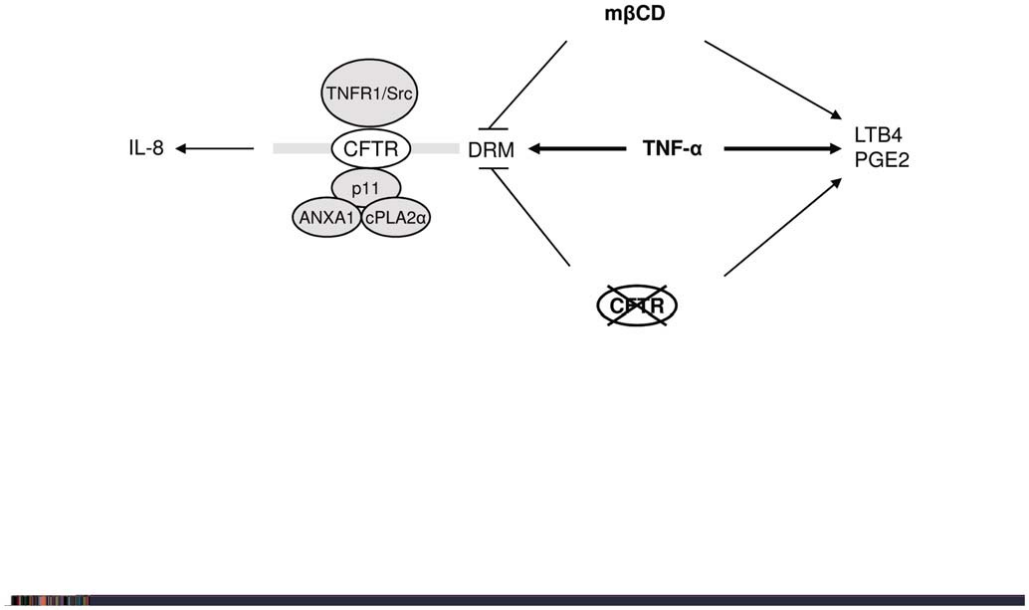
Hypothetical model linking CFTR/DRM interaction with cytokine and eicosanoid release. TNF-α exerts two effects that seem to be dissociated: eicosanoid release and IL-8 synthesis with the participation of DRM, in which CFTR, cPLA2α, ANXA1, TNFR1 and c-Src are transiently recruited. Both DRM destabilization (mβCD) and CFTR inhibition (Inh172) lead to increased eicosanoid release. However, they counteract in some conditions the other effect of TNF-α -in the case of Inh172, only at long term (12 h).

In conclusion, as summarized in Figure 9, acute TNF-α stimulation of Calu-3 cells leads to increased eicosanoid and IL-8 release, the latter relying on the integrity of DRM, which may contain a putative CFTR/cPLA2α/ANXA1/p11 complex. Both membrane destabilization by mβCD and CFTR inhibition, also favor eicosanoid synthesis independently of TNF-α, but tend to counteract the effect of this cytokine on DRM recruitment in certain conditions (Fig. 9). Our results show that the signaling events leading to eicosanoid and cytokine production are likely to take very different regulation routes, rendering inflammation treatment in CF especially complex. Considering our results and those of others, several questions await to be answered: (i) the nature of the sensing mechanism responsible for DRM recruitment, (ii) the role of CFTR function in recruitment, (iii) how DRM disruption and CFTR inhibition –without the participation of TNF-α- can activate the eicosanoid pathway, and (iv) the functional dynamics of the putative CFTR/ANXA1/cPLA2α/p11 complex, maybe including TNFR1/Src (Fig. 9). Our findings may contribute to a better understanding of membrane integrity and inflammation in CF, and a better knowledge of the CFTR interactome.
